# Herbosomal nanocarriers using natural-origin surfactants: a quercetin-based strategy for Alzheimer’s disease and oxidative-stress–driven neurodegeneration

**DOI:** 10.1038/s41598-026-53290-0

**Published:** 2026-05-26

**Authors:** Mostafa Okda, Soha M. El-Masry, Maged Wasfy Helmy, Haidy Abbas

**Affiliations:** 1 Department of Pharmaceutics, Faculty of Pharmacy, Damanhour University, Al Kornish, Damanhour, 22511 Egypt; 2 Department of Pharmacology and Toxicology, Faculty of Pharmacy, Damanhour National University, El-Bahira, Egypt

**Keywords:** Quercetin, Alzheimer’s disease, Herbosomes, Tween, Cocamidopropyl betaine, Biochemistry, Biotechnology, Chemistry, Drug discovery, Neuroscience

## Abstract

Alzheimer’s disease (AD) is a progressive neurodegenerative disorder characterized by oxidative stress, neuroinflammation, and cholinergic dysfunction. Quercetin (QUE) is a multifunctional flavonoid with potent antioxidant and anti-inflammatory effects and proven neuroprotective, anticancer, antimicrobial, and hepatoprotective potential. However, its therapeutic translation, particularly in the management of Alzheimer’s disease, is severely limited by low aqueous solubility, low bioavailability, and rapid metabolism. The current study aims to develop QUE-loaded herbosomes as an advanced phytophospholipid delivery system for AD treatment, with a focus on replacing the synthetic surfactant Tween 80 with natural-origin betaine surfactants to overcome the drawbacks of poor biocompatibility and chronic toxicity associated with conventional surfactants. QUE herbosomes were developed using the thin-film hydration method and evaluated for physicochemical characteristics, stability, and in vitro release behavior. Formulation variables were optimized to obtain herbosomal systems with favorable nanoscale properties and sustained drug release. DSC and FTIR analyses confirmed successful incorporation of quercetin within the vesicular structure. Compared with QUE suspension, the optimized QUE herbosomal formulations (F5 &F6) showed significantly higher effect in aluminum chloride-induced AD as evidenced by Behavioral testing, biochemical, and Histopathological analyses. These findings suggest that the developed QUE herbosomes with natural-origin surfactants offer a safe and biocompatible alternative to synthetic surfactant herbosomes, improving therapeutic outcomes in AD and holding promise for other oxidative stress-related neurodegenerative conditions.

## Introduction

Alzheimer’s disease (AD) is the most prevalent type of dementia affecting around 57 million people globally, of which 60–70% have AD (WHO 2025). AD starts with pathophysiological changes in the brain before any clinical signs are observed^1^. These changes include the accumulation of beta-amyloid plaques, which interfere with neuronal communication, and the dysregulation of Tau proteins, which interfere with neuronal cytoskeleton stability, neurite growth, axonal transport, and cell proliferation, considered mainsigns of the disease^2^. At the early stages of AD, hippocampal regions of the brain, which are responsible for memory formation, are affected with the disease progression, neuropathology becomes more widespread, resulting in a reduction of brain mass up to 35%, and the patients experience increasing disability and cognitive impairment during the disease progression^3^.

One of the main obstacles in AD management is the successful delivery of drug molecules to the brain across the blood–brain barrier (BBB). Although the BBB is crucial for protecting the brain from neurotoxins, it is also considered a significant obstacle that hinders drug delivery to the brain and needs to be overcome^4^.

Quercetin (QUE) is a naturally occurring flavonoid with a variety of therapeutic applications, including antioxidant, anti-inflammatory, neuroprotective, anticancer, antimicrobial, and hepatoprotective activity^5^. The antioxidant and anti-inflammatory effects of QUE are responsible for its great potential as a neuroprotective by reducing neuronal cell death, protein oxidation, and lowering the accumulation of beta-amyloid plaques, thereby slowing down Alzheimer’s disease progression^6^. Although the great potential of QUE, low solubility, low bioavailability, and extensive metabolism limit the ability to take its advantages and limit its therapeutic use^7,8^.

Herbosomes have emerged as a promising novel delivery system designed to overcome the pharmacokinetic and pharmacodynamic limitations associated with QUE^9^. These complexes are formed by combining natural phytoconstituents with phospholipids, predominantly phosphatidylcholine, the principal component of cell membranes, which enhances the permeability of phytoconstituents across biological barriers. Notably, the choline head group of phospholipids can form hydrogen bonds with QUE, thereby improving entrapment efficiency, stability, and permeability^10,11^. Furthermore, the phospholipids used in herbosomes are characterized by their biocompatibility, biodegradability, low toxicity, and excellent tolerability within the human body (Ghasemiyeh et al. 2018). Achieving sustained physical and chemical stability of herbosomes formulation upon storage is essential. The development of a stable formulation requires the selection of an appropriate surfactant at an appropriate concentration, which reduces surface tension and particle aggregation^12^. Furthermore, the formulation composition signifucantly influences its safety upon administration and permeability into the cells^13^.

Traditionally, non-ionic surfactants, such as Tween 80, have been widely used in nano-formulations due to their effective emulsifying and stabilizing properties, which are also considered the gold standard in preparations that cross the BBB^14^. The ability of Tween 80 to enhance brain delivery is due to its ability to adsorb apolipoprotein E (Apo E), making nanoparticles resemble low-density lipoproteins (LDL), which improves brain uptake via LDL receptor-mediated transcytosis^15^. Despite its widespread use as a non-ionic surfactant in pharmaceutical formulations, Tween 80 has been associated with several adverse effects^16^. It has been implicated in systemic reactions such as hypersensitivity, non-allergic anaphylaxis, and cutaneous manifestations including rash, as well as injection- and infusion-site complications such as pain, erythema, and thrombophlebitis^17,18^. Moreover, severe non-immunologic anaphylactic reactions have also been reported^19^. These safety concerns have driven the search for alternative surfactants of natural origin, such as betaine-based surfactants, which offer improved biocompatibility and reduced chronic toxicity compared to synthetic surfactants like Tweens and Spans^20,21^. In response to the limitations associated with synthetic surfactants, naturally derived alternatives are gaining increasing attention as safer and more sustainable options in pharmaceutical formulations. Cocamidopropyl betaine, a surfactant of green origin derived from coconut oil, exhibits unique properties, including biodegradability, biocompatibility, non-toxicity, and mildness, while also providing high stabilizing and solubilizing capacities. These attributes have enabled its successful application in stabilizing various nano-pharmaceutical formulations^22^. Furthermore, carboxybetaines are particularly suitable for biological applications due to their zwitterionic nature, which minimizes interactions with proteins. As a result, betaine-based surfactants offer significant advantages as effective, biocompatible stabilizers for the formation of kinetically stable nanoparticles, positioning them as strong competitors to conventional ionic and non-ionic surfactants in advanced drug delivery systems^22^. Despite the promising neuroprotective potential of QUE in Alzheimer’s disease, its clinical utility is limited by poor solubility, low bioavailability, and restricted blood–brain barrier permeability. The intranasal (nose-to-brain) route offers a non-invasive approach for direct drug delivery to the brain via the olfactory and trigeminal pathways, thereby bypassing the BBB and enhancing brain targeting. Accordingly, this study adopts the intranasal route to improve the brain delivery and therapeutic efficacy of QUE.

The current work aims to develop QUE herbosomes for the treatment of AD and to evaluate the feasibility of replacing Tween 80, a widely used synthetic surfactant known for its role in enhancing blood–brain barrier (BBB) permeability, with the naturally derived surfactant cocamidopropyl betaine. This strategy is intended to enhance brain targeting by bypassing the blood–brain barrier (BBB) via the intranasal route, while improving biocompatibility and reducing the potential toxicity associated with conventional surfactants. The efficacy of the developed formulas was then assessed in AlCl_3_-induced Alzheimer’s disease using histological and biochemical assessments.

## Materials and methods

### Materials

Quercetin powder was purchased from (Sigma-Aldrich, Germany). Lipoid ® S 75 was generously gifted by (Lipoid GmbH, Germany). Cholesterol (Sigma-Aldrich Chemie GmbH, Germany)., Tween 80 (oxford labchem,India). Methanol and chloroform of high HPLC grade (Fisher Scientific, UK). Propylene glycol (Piochem, Egypt), Cocoamidopropyl betain (Ecochem, Malaysia). Visking® dialysis tubing (MWCO 12,000–14,000) was purchased from (SERVA Electrophoresis GmbH, Germany), Aluminum chloride was purchased from (Loba Chemie Pvt. Ltd, Mumbai, India). Normal Saline 0.9% (ADWIC, Egypt). Formalin (PIOCHEM, Egypt). Acetylcholinesterase Activity Assay Kit (Catalog No: MAK119) from (Sigma-Aldrich, USA). Amyloid-Beta Aβ42 (Catalog No: ER0775) from (Wuhan Fine Biotech Co., Ltd,China). Rat phosphorylated Tau 181 (PT-181) ELISA Kit (catalog NO: MBS9905658) from (MyBioSource Inc, USA). Rat Nuclear Factor Kappa B p65 (NF-kB p65) ELISA Kit (catalog NO: MBS2505513) from (MyBioSource Inc,USA). Total Antioxidant Capacity Assay Kit (Cat. No. ab65329) was obtained from (Abcam limited, UK).

## Methods

### Preparation of QUE herbosomes

QUE-loaded herbosomes were prepared using the thin-film hydration method with different molar ratios of QUE, Lipoid®S75, and cholesterol, as described previously by Telange et al.^23^. The composition of the investigated herbosomal preparations was summarized in Table 1. Briefly, QUE, Lipoid®S75, and cholesterol were solubilized in a 1:1 (v/v) mixture of chloroform and methanol in a round-bottom flask and sonicated for 2 minutes. Then, the mixture was refluxed under magnetic stirring for 2 h at 50 °C (120 rpm) to ensure adequate complexation between QUE and phospholipid. The resultant solution was subsequently subjected to rotary evaporation (Buchi R-100, Switzerland) at 50 °C and 120 rpm under reduced pressure (~300 mbar) for 2 h to ensure complete solvent removal and formation of a uniform thin lipid film. These conditions were applied as fixed processing parameters and are consistent with commonly reported temperature ranges (45–50 °C) for stable phospholipid-based systems (Šturm et al. 2021). QUE herbosomes were prepared through a self-assembly method^24^. Briefly, the resulting thin film was hydrated with 10 mL of phosphate buffer (pH 7.4) in a rotary evaporator at 50 °C and 120 rpm for 1 hour. The resultant herbosomal dispersion was then subjected to size reduction using bath sonication (Selecta, Italy) for 20 minutes.

**Table 1:** Composition of different quercetin herbosmal preparations, molar ratio.

Formula	Quercetin: Lipid molar ratio	Cholesterol (% w/w)	Tween 80 (% w/w)	Cocoamidopropyl betaine (% w/w)
F1	1:1	0	0	0
F2	1:2	0	0	0
F3	1:2	1	0	0
F4	1:2	0.2	0	0
F5	1:2	0.2	10% w/w of lipid	0
F6	1:2	0.2	0	10% w/w of lipid

### In vitro characterization of QUE herbosomes

#### Vesicle size (VS), polydispersity index (PDI), and zeta potential (ZP)

Nanoparticle size analyzer (Nanotrac wave ǁǁ, USA) has been used to measure the vesicle size, PDI, and ZP of the prepared herbosomal dispersions. Samples from each formulation were diluted 100-fold in deionized water prior to analysis. The results were shown in triplicate as a mean ± SD.

#### Percent of entrapment efficiency (EE%)

A 1 ml volume of herbosomal dispersions equivalent to 1 mg of QUE was centrifuged in a cooling centrifuge (Herolab, Unicen HR, Germany) at 15,000 rpm for 1 h at 4 °C. The supernatant was then separated and filtered using a 0.45 µm Millipore syringe filter. Unentrapped QUE in supernatant was determined from the calibration curve at 373 nm. The percent of entrapment efficiency was determined using the following equation.

$${\mathrm{EE}}\% = \frac{{{\mathrm{W}}\left( {\text{total QUE content}} \right){-}{\mathrm{W}}\left( {\text{unentrapped QUE}} \right)}}{{{\text{W }}({\text{total QUE content}})}} \times {1}00$$

#### In vitro release of QUE herbosomes

The in vitro release of QUE from QUE aqueous suspension and different QUE herbosomal dispersions was determined by the dialysis bag method. Briefly, Aliquots (2ml) of herbosomal dispersions equivalent to 2 mg of QUE were filled into a dialysis bag and then immersed in a breaker containing 50 mL of release medium consisting of phosphate buffer saline (PBS, pH 7.4) containing 30% v/v propylene glycol (PG), selected based on preliminary studies to maintain sink conditions for poorly soluble QUE (Kendre et al. 2014; Aboud et al. 2016). The system was maintained at 37 ± 1 °C and 100 rpm. At predetermined intervals (0.5–24 h), aliquotes of 4 ml of the release media were periodically withdrawn at 0.5, 1, 2, 3, 6, 8, and 24 h and compensated with fresh release media to preserve sink conditions. The withdrawn samples were filtered using a 0.45 µm Millipore syringe filter and then measured spectrophotometrically at 373 nm^25^.

#### QUE herbosomes release kinetics

Different kinetic models, including zero-order, first-order, Higuchi’s model, Korsmeyer-Peppas, and Hixson–Crowell model, were used to study QUE release kinetics from herbosomal dispersions. The kinetic release models were determined using DDSolver software, which utilized data from in vitro drug release studies to calculate the adjusted R^2^, employed to identify the best-fitting kinetic model.

#### Transmission electron microscopy (TEM)

Morphological evaluation of the chosen formulas (F5& F6) was performed using TEM (JEOL JEM-1400, Japan). Briefly, a drop of herbosomal dispersion was added to a carbon-coated copper grid and allowed to air-dry at room temperature prior to examination at 80 kV.

#### Differential scanning calorimetry (DSC)

Samples of pure QUE, lipoid® S75, cholesterol, their physical mixture, and the selected QUE formulations (F5&F6) were analyzed for their thermal behavior using a differential scanning calorimeter (LINSEIS STA PT-1000, China). Approximately 5 mg of each sample was added to aluminum crucibles and heated under constant nitrogen gas, adjusted at a flow rate of 30 mL/min, across a temperature range of 30–400 °C with a constant heating rate of 10 °C/min.

#### Fourier transformation infrared spectroscopy (FTIR)

To investigate possible interactions, FTIR spectra were recorded for pure QUE, lipoid® S75, cholesterol, Tween 80, cocamidopropyl betaine and physical mixture (pure QUE, lipoid® S75, cholesterol), and the selected QUE formulations (F5and F6). The spectra were obtained using an Agilent Cary 630 FTIR spectrometer (Malaysia) within the wavenumber range of 4000–700 cm^−1^.

#### Stability studies

To evaluate the physical stability of the selected formulations (F5 and F6), samples were stored at 4 °C for three months. Then, their Vesicle size, ZP, and EE% were compared with those of freshly prepared formulations (month zero).

### In vivo studies

#### Experimental animals

**Fig. 1:**
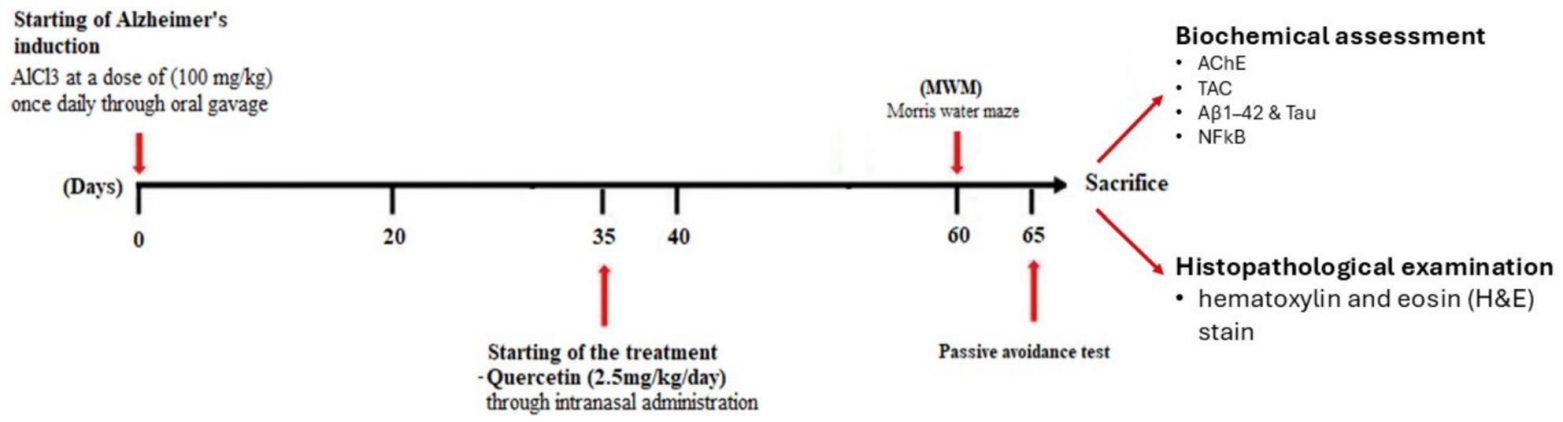
Schematic representation of the experimental timeline and dosing schedule.

Forty-two adult male Wistar albino rats (10–12 weeks of age, weighing 180–210 g) were provided by the animal House of the Faculty of Medicine, Alexandria University. Therats were housed six per cage in polypropylene cages (75 cm × 40 cm × 25 cm) under standard conditions (temperature 25 ± 3 °C and humidity 50 ± 5% with a 12-h light/12-h dark cycle and free access to water and food*)* and allowed to adapt for one week before the experiment to ensure acclimatization. All experiment procedures were carried out during the daytime (10:00 am − 6:00 pm). The experimental timeline and dosing schedule are summarized in Fig. 1. Ethical approval for the study was obtained from the Research Ethics Committee, Faculty of Pharmacy, Damanhour University, Egypt (Approval No. 619PT10). The work was performed in compliance with the *Guide for the Care and Use of Laboratory Animals* (NIH Publication No. 8023, revised 1978).

#### Induction of Alzheimer’s model (AD)

An Alzheimer’s disease-like model was induced by administration of a solution of AlCl3 once daily through oral gavage at a dose of 100 mg/kg body weight for 35 days^26^. All rats were assigned to Alzheimer’s disease-like model inductions, except for Group 1, which served as the negative control group and received only oral normal saline.

#### Treatment protocol

After the induction of Alzheimer’s disease-like model using AlCl3 for 35 days, 50 μl of different treatments were administered through intranasal administration using a micropipette at a dose equivalent to 2.5 mg QUE/kg/day for 30 days^27^. Briefly, rats were placed in a supine position to allow the treatment to reach the olfactory region, and 50 μl of different treatments was slowly applied into the right nostril^28^. Treatment groups were as follows:

Group 1: (negative control group): administered normal saline.

Group 2: (AlCl3-induced positive control group): administered normal saline.

Group 3: administered QUE suspension.

Group 4 administered a blank formulation prepared with Tween 80 (Blank F5).

Group 5: administered blank formulation prepared by cocamidopropyl betaine (Blank F6).

Group 6: administered F5 formulation.

Group 7: administered F6 formulation.

#### Behavioral testing

##### Morris water maze (MWM) test

The MWM test was used to measure memory and learning ability in rats across the seven groups. Rats were directed to swim to a hidden board in a circular black pool filled with water at 25 ± 2 °C, to a depth of 40 cm. The pool was sectioned into four sections, and a board measuring 10 cm in diameter was submerged 2 cm under the surface of water in one of the four sections (the goal quadrant) where it was kept throughout all training sessions. Starch was used to make the water opaque, and a visual cue was utilized to aid the rats in identifying their location. The experiment consisted of four training sessions, followed by a probe test on the fifth day. Each training session consisted of four rounds for each rat. In each round, the rat was released from a different section and given 90 s to locate the hidden board. If the rat failed to find the hidden board within 90 s, it was manually guided to it and placed on it for 20 s^29^. In the probe test, the board was removed, and the escape latency time, which is the time required for each rat to locate the board position, was recorded^30^.

##### Passive avoidance test

The passive avoidance apparatus consists of 2 compartments (light and dark compartments). The two compartments were separated by a wall with an exit door, and the floor of the dark compartment contains stainless steel rods that deliver 0.5 mA electric shocks for 3 s^31^. Animals were tested one day before sacrifice, following a one-day training period. In a training session, rats were individually transferred to the light compartment, and an electrical foot shock was administered when they entered the dark compartment. Then, the rats were immediately removed from the compartment and returned to their cages. The test session was conducted 24 h after the training session, but without administering the foot shock. The time between placement of the rats in the light compartment and entry into the dark compartment was defined as the step-through latency time^32^. When rats did not move from the light to the dark compartment within 180 s, the step-through latency time was recorded as 180 s^33^.

#### Biochemical testing

##### Tissue preparation

The rats were anesthetized using thiopental (50mg/kg of body weight) (Helmy et al. 2014). Then the rats were euthanized by decapitation. The whole brain was carefully removed from the skull and separated into two halves, and the hippocampus of each half was separated^34^. One hippocampal half was washed with normal saline and stored at −80 °C for further biochemical assessment. The other half was kept in 10% formalin for histological examinations.

##### Biochemical assessment

Rat hippocampal tissue was weighed and homogenized using PBS at pH 7.4. Then, it was centrifuged at 1000 rpm using a cooling centrifuge for 15 minutes at 4 °C. The supernatant was removed and stored at −80 °C^7,35^ for biochemical assessment of the following parameters: total antioxidant capacity (TAC), Nuclear Factor Kappa B (NF-κB), Acetylcholinesterase (AChE), β-amyloid, and Tau protein.

###### Total antioxidant capacity estimation

The total antioxidant capacity was measured using a colorimetric assay kit according to the method supplied with the kit. The absorbance was determined spectrophotometrically at 570 nm, and the resulting absorbance values were used to construct a calibration curve for determining sample TAC, expressed in mmol/mg protein.

###### Acetylcholinesterase (AChE) estimation

Acetylcholinesterase activity was measured using an AChE assay kit, and the procedures were performed according to the method supplied with the kit. The absorbance was determined spectrophotometrically at 412 nm, and the resulting absorbance values were used to construct a calibration curve to determine ACHE activity, expressed as U/mg protein.

###### Amyloid Beta (1–42) and Tau estimation

Aβ1–42 and rat phosphorylated Tau 181(PT-181) ELISA kits (colorimetric) were used to measure Amyloid Beta (1–42) and rat phosphorylated Tau 181(PT-181) concentration according to the method supplied with the kit. The absorbance values were determined spectrophotometrically at 450 nm and used to construct a calibration curve to determine the concentrations of Aβ1–42 and Tau proteins, expressed in pg/mg protein.

###### Nuclear Factor Kappa B-p65 estimation

Rat Nuclear Factor Kappa B-p65 (NFkB-p65) ELISA Kit (colormetric) was utilized to measure NFKB-p65 Concentration according to the method supplied with the kit. The absorbance values were measured spectrophotometrically at 450 nm and used to construct a calibration curve for determining (NFkB-p65) concentration, expressed in ng/mg protein.

#### Histopathological examination

For preparing rat hippocampus histological sample slides, the rat hippocampus was kept in 10% formalin for 24 hours, then rinsed with water. Next, the samples were dried using a gradual serial dilution of alcohol. Then, the samples were cleared using xylene, and finally, they were embedded in paraffin blocks. For light microscopy examination, paraffin blocks were sectioned to 4-μm thickness using a microtome (SLEE, CUT 5062, Germany), then the sections were stained using hematoxylin and eosin (H&E) stain^36^.

#### Statistical analysis

All statistical analyses were conducted using GraphPad Prism® version 10 (GraphPad Software, San Diego, CA, USA). Data were expressed as mean values ± standard deviation (SD). To compare the properties of the prepared formulations, a t-test was employed. In contrast, the outcomes of *in-vivo*, biochemical, and histopathological evaluations were analyzed using one-way analysis of variance (ANOVA), followed by Tukey’s post hoc test. Statistical significance was defined as *p* < 0.05.

## Results and discussion

### Preparation of QUE herbosomes

The thin-layer evaporation method was chosen because of its simplicity and ability to produce relatively stable nanoparticles. In addition, Preparation conditions, such as temperature, hydration media, and rotation speed, can be modulated to produce particles that meet the desired criteria. The optimal rotation speed was 120 rpm, forming a uniform, thin film, which facilitated the homogeneous production of herbosomes. The selection of a hydration medium basically depends on the final dosage form and the route of administration. In this study, PBS (pH 7.4) was used due to the safety concerns associated with intranasal administration. Moreover, salts present in the buffer facilitate the self-assembly of lipids into a vesicular system^37^.

### In vitro characterization of QUE herbosomes

#### Determination of vesicle size, PDI, and ZP

Vesicle size, PDI, and ZP results are summarized in Table 2. The mean Vesicle sizes of QUE herbosomes ranged from 289.4±4.83 nm (F1) to 194.3±2.62 nm (F6). Results showed that the addition of surfactants (Tween 80 or cocamidopropyl betain) caused a reduction in Vesicle size due to steric repulsion exerted by surfactant, minimizing vesicular aggregation^38^. Additionally, the incorporation of cholesterol significantly influenced Vesicle size. The optimal Vesicle size reduction was achieved at a 0.2 molar ratio of cholesterol. This observation may be due to cholesterol’s ability to enhance the bilayer’s hydrophobicity, thereby reducing the surface free energy and, consequently, reducing vesicle size^39^. Increasing the cholesterol content beyond a 0.2 molar ratio, the Vesicle size increased. This effect is likely attributed to cholesterol-induced lipid ordering and increased membrane thickness, as evidenced by tighter phospholipid packing and increased membrane rigidity^40,41^. The addition of cholesterol at an 80:20 ratio caused a significant size reduction compared to the cholesterol-free formulation. These findings indicate that incorporating cholesterol into the lipid bilayer enhances the system’s stability, achieving the desired increase in lipid order^41^. Comparing the effect of the two surfactants (Tween 80 and cocamidopropyl betaine), there is a relationship between the degree of lipophilicity of surfactants and the resulting Vesicle sizes, and it was reported that higher HLB values of the surfactant the larger vesicles will be obtained^42^. The HLB values of Tween 80 and cocamidopropyl betaine were 15 and 13.4, respectively, which clarifies the lower Vesicle size obtained with cocamidopropyl betaine.

**Table 2:** Physico-chemical characterization of QUE herbosomes (n = 3; mean ± SD).

Formula	Vesicle size (nm)	PDI	ZP (mV)	EE (%)
F1	289.4 ± 4.83	0.228 ± 0.039	− 23.90 ± 2.816	83.74 ± 0.45
F2	283.7 ± 6.54	0.191 ± 0.061	− 26.83 ± 3.33	88.88 ± 0.72
F3	245 ± 2.36	0.17 ± 0.023	− 33.53 ± 2.91	81.74 ± 1.26
F4	231.23 ± 4.46	0.243 ± 0.076	− 29.03 ± 1.45	92.31 ± 0.33
F5	207.63 ± 6.63	0.228 ± 0.012	− 28.8 ± 2.09	90.11 ± 0.98
F6	194.3 ± 2.62	0.258 ± 0.046	− 30.90 ± 2.01	91.0 ± 0.39

Polydispersity index (PDI) is a critical factor that reflects the uniformity of the size distribution of nanocarriers. According to Kaur et al.^43^, PDI values exceeding 0.7 denote a broad Vesicle size distribution, which may negatively impact formulation stability and reproducibility. The PDI results of the prepared QUE herbosomes ranged from 0.17 ± 0.023 to 0.258 ± 0.046. This indicates that the prepared QUE herbosomes possess a narrow and consistent size range.

ZP is used to demonstrate the surface charge that indicates the stability of the formed herbosomes. Higher ZP values indicate a lower aggregation tendency of vesicles^44^. Zeta potential of all formulations was negative, ranging from −23.90±2.816 mV for (F1) to −33.53±2.91 mV for (F3), indicating a good stability profile for all prepared formulations. The values of ZP slightly increased with increasing cholesterol concentration, which indirectly reflects the stability of herbosomes. Cholesterol incorporation increases the inter-head group distance within the phospholipid bilayer, thereby promoting hydrophobic stabilization. Additionally, cholesterol alters the lipid packing order and bilayer thickness, facilitating hydrogen bonding interactions and influencing the overall charge distribution on the herbosome’s surface^45^. Compared to Tween 80 (F5), (F6) containing cocamidopropyl betaine exhibited a non-significant reduced Vesicle size. At the same time, PDI and zeta potential values showed no notable differences, suggesting that the substitution maintained colloidal stability despite the change in surfactant.

#### Entrapment efficiency

The optimization of entrapment efficiency (% EE) is crucial because it greatly influences drug dosing and its release characteristics^46^. In the study of entrapment efficiency (EE%) of QUE herbosomes, two primary factors were identified as influential: the drug-to-lipid molar ratio and the lipid content. It was observed that a higher lipid ratio significantly enhanced the entrapment of water-insoluble drugs^47^. Visual examination of the formulations with a ratio less than 1:2 revealed the formation of precipitate shortly after hydration. This can be attributed to two main factors: incomplete entrapment of QUE and an inadequate amount of lipid for the entrapment of QUE. The optimal entrapment of QUE was achieved with a molar ratio of QUE to lipid of 1:2. This result aligns with the findings by^37^. The second factor affecting EE% was the cholesterol molar ratio. The interaction between cholesterol and phospholipid, which helps phospholipid molecules to be more tightly arranged and increases the hardness and thickness of the complex membrane, may be the reason why QUE entrapment efficiency increased when the molar ratio of CH: lipid equaled 0.2:1. Consequently, the herbosomes might be shielded from drug leakage. In contrast, when the molar ratio of CH: lipid increased to 1:1 (F3), the EE% decreased compared to that of other samples (81.74% ± 1.26). It can be explained that the elevated cholesterol concentration forced the displacement of QUE from the phospholipid bilayer membrane due to competition for its position, thereby decreasing its incorporation into herbosomes^48^. The entrapment efficiency (EE%) of QUE herbosomes stabilized with cocamidopropyl betaine was slightly higher than that of Tween 80 formulations, with no statistically significant variation observed (*P* > 0.05). This may be due to the amphiphilic nature of cocamidopropyl betaine, which prompts QUE incorporation within the vesicular structure^22^.

#### In vitro drug release of QUE

**Fig. 2:**
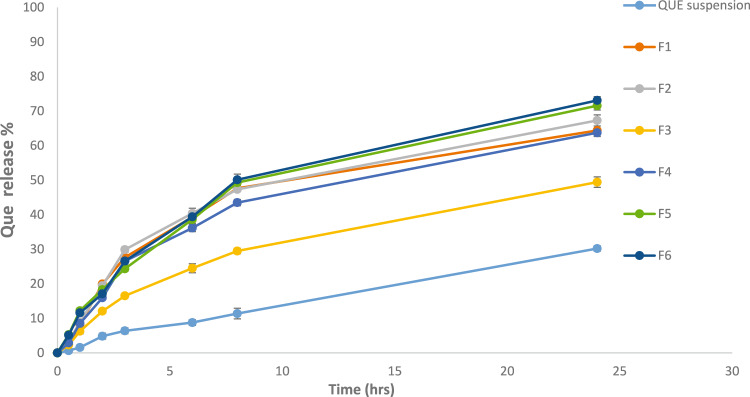
In vitro drug release of QUE herbosomes formulations and QUE suspension in PG/PBS 30% (v/v) at 35 ± 1°C. Results are means ± SD, (n = 3).

The release of QUE from various formulations (F1–F6) and QUE suspension was evaluated over 24 hours using PG/PBS (30% v/v, pH 7.4) as the release medium, which was selected based on preliminary studies for optimal release. The percentage of drug release of QUE from herbosomal formulations compared to free QUE suspensions is revealed in Fig. 2 and Table 3. All herbosomal formulations demonstrated a marked enhancement in QUE release compared to the QUE suspension, which exhibited the lowest percent of release at all time points (30.21 ± 0.76% at 24 hours).

**Table 3:** In vitro drug release of QUE from herbosomes and QUE suspension (n = 3; Mean ± SD).

Time (hours)	QUE suspension	F1	F2	F3	F4	F5	F6
QUE release %							
0.5	0.65 ± 0.58	2.50 ± 0.34	3.33 ± 0.42	2.31 ± 0.61	2.88 ± 0.22	5.30 ± 0.60	5.14 ± 0.46
1	1.58 ± 0.62	8.25 ± 0.24	8.87 ± 0.54	6.28 ± 0.84	8.53 ± 0.76	12.21 ± 0.66	11.59 ± 0.50
2	4.85 ± 0.86	19.94 ± 0.41	19.46 ± 0.51	12.09 ± 0.39	15.96 ± 0.58	18.32 ± 0.76	17.12 ± 0.86
3	6.39 ± 0.84	27.65 ± 0.33	29.89 ± 0.80	16.52 ± 0.66	26.58 ± 0.30	24.34 ± 0.73	26.58 ± 1.15
6	8.78 ± 0.83	39.31 ± 0.34	40.39 ± 1.49	24.51 ± 1.07	36.17 ± 1.31	38.61 ± 1.20	39.41 ± 1.45
8	11.39 ± 1.54	47.62 ± 0.80	47.33 ± 0.42	29.50 ± 0.91	43.50 ± 0.77	49.30 ± 0.95	50.12 ± 1.64
24	30.21 ± 0.76	64.39 ± 1.08	67.29 ± 1.60	49.43 ± 1.12	63.74 ± 1.52	71.57 ± 1.24	73.09 ± 1.08

The composition of the herbosomes significantly influenced drug release. F1 and F2, which lacked cholesterol (QUE: Lipoid S75 at ratios of 1:1 and 1:2, respectively), showed a lower percent of release than cholesterol-containing formulations except for F3. The addition of cholesterol at 0.2% w/w in F4, F5, and F6 (all with a 1:2 QUE: Lipoid S75 ratio) enhanced drug release, highlighting cholesterol’s role in stabilizing vesicular structure and modulating release kinetics. However, increasing the cholesterol content to 1% w/w F3 resulted in a slower release pattern (49.43 ± 1.12% at 24 hours), likely due to increased hydrophobic interactions and steric hindrance between QUE and the lipid bilayer, which can hinder entrapment efficiency and limit drug release^49^.

The inclusion of surfactants further improved QUE release. Both F5 (containing Tween 80, a non-ionic synthetic surfactant) and F6 (containing cocamidopropyl betaine, a natural surfactant) exhibited the highest release rates, with percent drug releases of 71.57 ± 1.24% and 73.09 ± 1.08% at 24 hours, respectively. This enhancement is attributed to surfactants’ ability to increase drug solubility in aqueous medium^20,21^. Notably, the release profile of F5 was nearly identical to that of F6 with no statistically significant difference (*P*>0.05). This suggests that substituting Tween 80 with cocamidopropyl betaine maintains optimal QUE release while potentially improving biocompatibility and safety, owing to the amphiphilic structure of cocamidopropyl betaine, which enhances drug solubilization^22^. These results demonstrate the importance of both cholesterol inclusion at an optimal concentration and an optimized lipid-to-drug ratio. Most importantly, they demonstrate the feasibility of replacing synthetic surfactants like Tween 80 with natural alternatives such as cocamidopropyl betaine, without compromising formulation performance or drug-release efficiency.

#### Release kinetics

The drug release profile from herbosomal formulations was fitted to different release kinetics models, zero-order, first-order, Higuchi, Korsmeyer-Peppas , and Hixson-Crowell. Table 3 presents the coefficient of determination (R^2^) for various release kinetic models. The best-fit model was determined based on the highest R^2^^50^. The Korsmeyer-Peppas model demonstrated the best fit for all formulations (F1-F6), with release exponent (n) values ranging from 0.45 to 0.89, indicating an anomalous (non-Fickian) diffusion-controlled release mechanism^51^ (Elmasry et al. 2024). This suggests that diffusion processes contribute to QUE release from the formulations^52^.

**TEM**

**Fig. 3:**
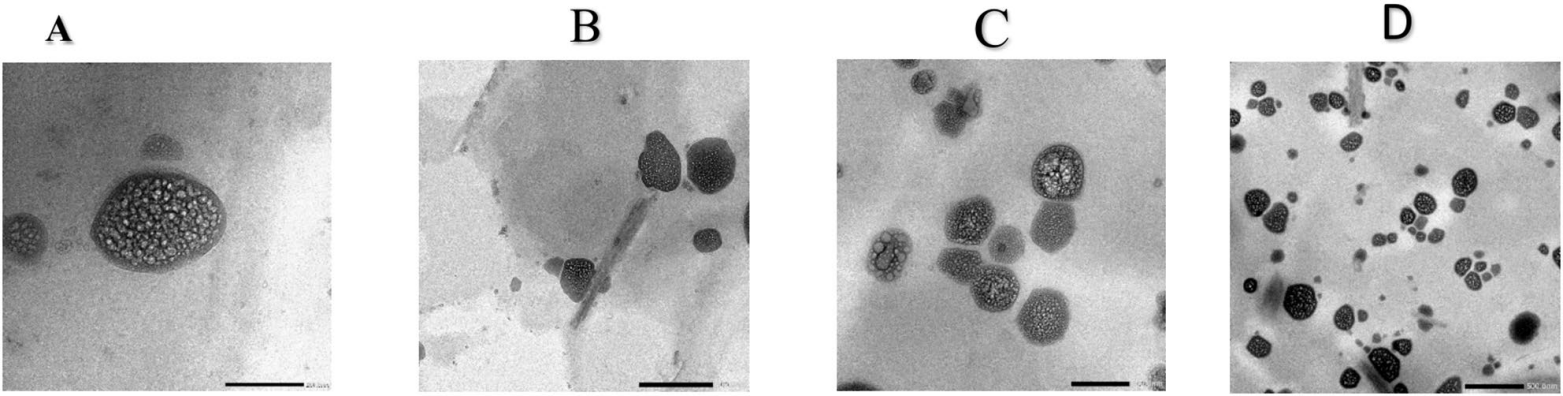
TEM micrographs of Formula F5 (**A**, **B**) and Formula F6 (**C**, **D**) showing the morphology and structural characteristics of the vesicles. (scale bar = 200 nm,500 nm).

The TEM examination of the optimized QUE formulations (F5 and F6) is shown in Fig. 3. Examination showed that both herbosomes (F5 & F6) have spherical morphology with small, uniform particle size with minimal aggregation tendency. This observation is in agreement with the particle size and PDI results measured by dynamic light scattering, supporting the consistency and stability of the formulated vesicles (Table 4).

**Table 4:** Drug release kinetics of QUE from herbosomes formulations using different release models.

Release models	Kinetic parameter	F1	F2	F3	F4	F5	F6
Zero order	R2	0.4621	0.4809	0.7222	0.5664	0.5937	0.6042
First order	R2	0.8416	0.8655	0.8773	0.8725	0.9297	0.9397
Higuchi	R2	0.9299	0.9412	0.9728	0.9516	0.9689	0.9652
Korsmeyer-Peppas	R2	**0.9305**	**0.9416**	**0.9837**	**0.9521**	**0.9700**	**0.9668**
	N	0.486	0.489	0.566	0.514	0.520	0.524
Hixson Crowell	R2	0.7417	0.7703	0.8311	0.7910	0.8576	0.8718

Bold values indicate the best-fi t kinetic model, selectedbased on the highest correlation coeffi cient (R²) value.

#### Differential scanning calorimetry (DSC)

**Fig. 4:**
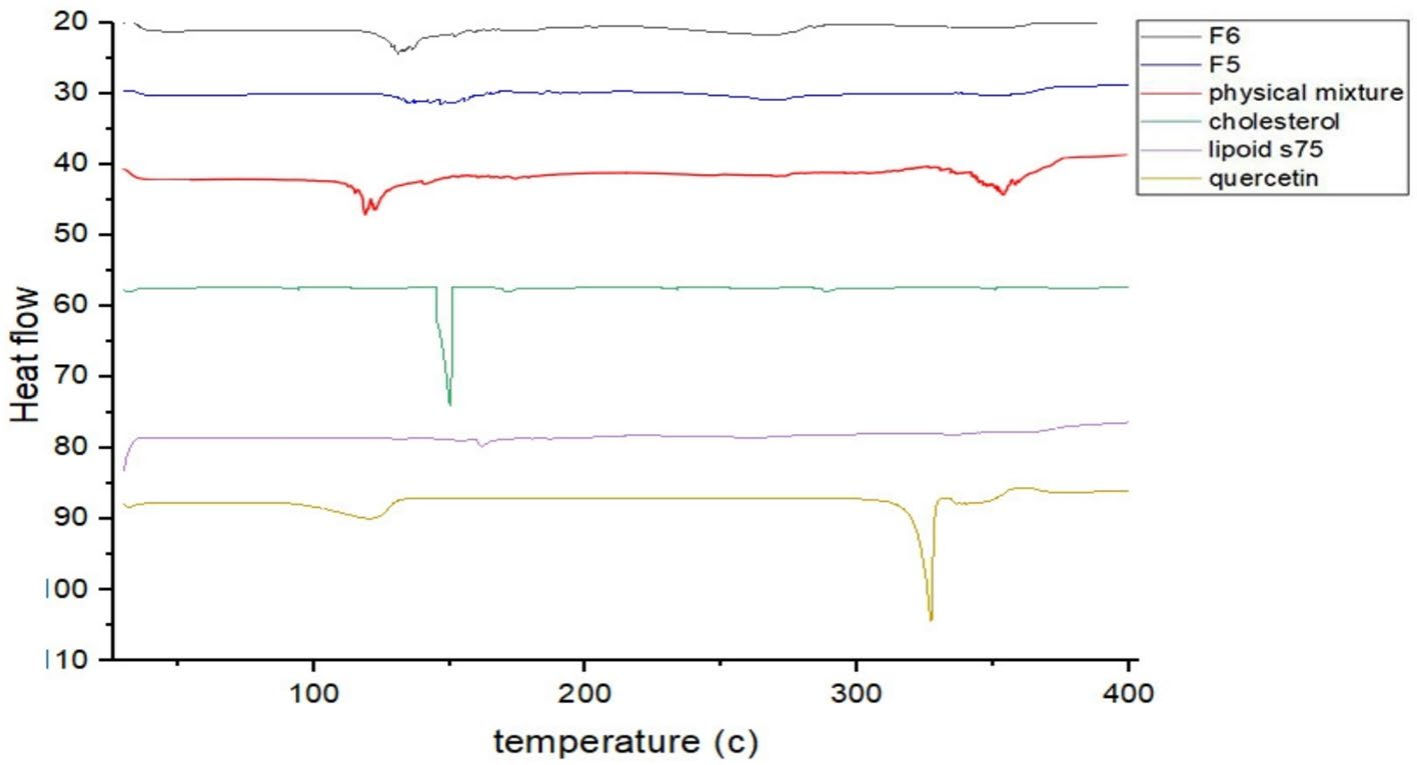
DSC thermogram of pure quercetin, lipid s75, cholesterol, physical mixture, quercetin-loaded Tween herbosomes (F5) and quercetin-loaded cocamidopropyl betaine herbosomes (F6).

DSC analyses of QUE and lyophilized-optimized formulations were conducted to determine the degree of QUE crystallinity after its incorporation into herbosomes^53^. DSC serves as a reliable analytical technique for detecting potential interactions between drugs and formulation excipients. It is also widely employed to examine the thermal behavior of crystalline substances, including drugs incorporated into various nano systems (Maiti et al. 2007; Trasi et al. 2020). Furthermore, it can help determine the localization of a drug within nanocarriers, offering insights into whether the drug is encapsulated within the lipid bilayer or dispersed in the aqueous compartment (Gokce et al. 2012; Akbarzadeh et al. 2013). Figure 4 shows thermograms of pure QUE, lipidoid s75, cholesterol, their physical mixture, and QUE-loaded herbosomes formulations (F5 & F6). The thermal profile of pure QUE exhibited two distinct endothermic peaks: one at 118 °C, associated with moisture loss, and the second at 325 °C, reflecting sharp thermal degradation and confirming its crystalline nature. These results are consistent with previously reported data^54,55^.

The thermal analysis of the optimized formulations indicated successful incorporation of QUE into the lipid matrix. DSC thermograms showed the complete disappearance of QUE’s characteristic melting peak at approximately 325 °C, along with a downward shift in the cholesterol endotherm, indicating loss of crystallinity and molecular dispersion within the lipid matrix (Munot et al. 2023). These changes suggest hydrogen bonding formation between the (OH) groups of QUE and phosphatidylcholine polar head groups, facilitating flexible tail rotation and encapsulation efficiency (Lu et al. 2019; Hendawy et al. 2023). This interaction contributes to the structural integrity and stability of the resulting herbosomes. These findings were further supported by FTIR analysis.

#### Fourier transform infrared spectroscopy (FTIR)

**Fig. 5:**
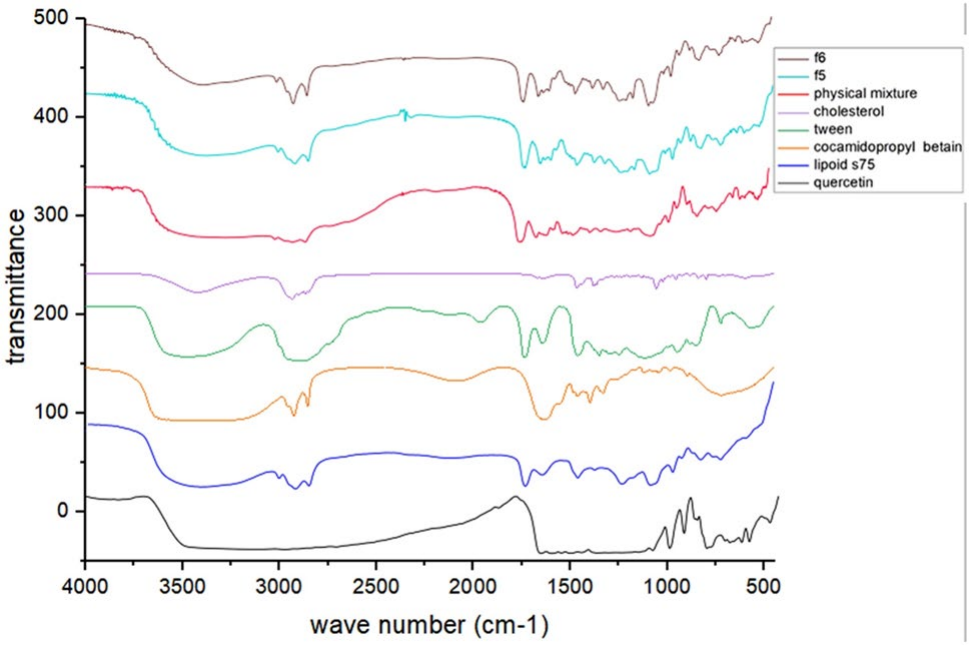
FTIR spectra of of pure quercetin, lipid s75, cholesterol, physical mixture, Tween80, cocamidopropyl betaine, quercetin-loaded Tween herbosomes (F5) and quercetin-loaded cocamidopropyl betaine herbosomes (F6).

The possible interactions between QUE and the formulation excipients were analyzed using FT-IR spectroscopy. Figure 5 illustrates the spectra of pure QUE, lipoid^®^ S75, cholesterol, Tween 80, cocamidopropyl betaine, and physical mixture, and the optimized formulations (F5&F6). The FT-IR spectrum of pure QUE exhibited distinct absorption bands at 3361 cm^−1^ (O–H stretching of hydroxyl groups), 1627 cm^−1^ (C=O stretching of carbonyl groups), 1597 cm^−1^ (C=C stretching of the aromatic ring), at 1242 cm^−1^ (O–H bending of phenolic groups), 1157 cm^−1^ (C–O–C stretching vibration) and at 995 cm^−1^ (aromatic C–H bending), which aligned with previously reported findings^56,57^. The spectrum of Lipoid^®^ S 75 displayed a broad absorption at 3309 cm^−1^ (O–H stretching), peaks at 2916 and 2854 cm^−1^ (C–H stretching of long-chain fatty acids), a sharp band at 1735 cm^−1^ (C=O stretching of fatty acid esters), 1234 cm^−1^ (P=O stretching of phospholipids), and 962 cm^−1^ (N^+^–(CH_2_)_3_ stretching). The FTIR spectrum of the physical mixture exhibited a superimposed pattern of both QUE and excipient peaks corresponding to each individual component present in the formulation. This observation suggests the physical coexistence of all materials without significant peak shifts or disappearance, except for minor changes associated with hydrogen bond formation.

FTIR analysis of F5 and F6 revealed slight reductions in intensity and shifts in specific peaks of QUE. Notably, the phenolic hydroxyl vibration band of QUE shifted from 3361 to 3294 cm⁻^1^, potentially indicating hydrogen bond formation. Additionally, the –P=O stretching peak of the phospholipid shifted from 1234 to 1126 cm⁻^1^ in the optimized formulation. These minor spectral changes suggest modifications in the vibrational functional groups of QUE, likely due to its successful incorporation into the formulation matrix via hydrogen bonding or physical entrapment, without the formation of new covalent bonds.

These spectral observations were strongly supported by previously conducted DSC analysis, which showed no significant changes in the thermal profiles of QUE, confirming the physical integrity and compatibility of the selected formulations (F5&F6).

#### Stability study

The physicochemical stability of formulations F5 and F6 was assessed after storage for three months at 4 °C by remeasuring VS, ZP, PDI, and %EE. As shown in Table 5, no statistically significant changes were observed in any of the measured parameters, demonstrating that both formulations maintained their physical integrity throughout the storage period. Regarding Vesicle size, the Vesicle size was increased from 207.63 ± 6.63 nm to 220.3 ± 4.16 nm and from 194.3 ± 2.62 nm to 205 ± 6.05 nm for F5 and F6, respectively, and the zeta potential decreased from −28.8 ± 2.09 to -26.83 ± 0.746and from −30.90 ± 2.01 to −28.95 ± 1.39 for F5 and F6, respectively, entrapment efficiency was decreased from 90.11 ± 0.98% to 88.07 ± 0.76% and from 91.00 ± 0.39% to 89.26 ± 1.03% for F5 and F6 respectively. These results collectively demonstrate the favorable storage stability of QUE herbosomes (F5 and F6) under refrigerated conditions.

**Table 5:** Stability study indicating Vesicle size, PDI, ZP, and EE% of the optimized formulations (F5) and (F6) after storage for three months at 4 °C.

Parameters		Vesicle size (nm)		PdI		ZP (mV)		EE (%)	
Formulation		F5	F6	F5	F6	F5	F6	F5	F6
Storage time (Months)	Zero	207.63 ± 6.63	194.3 ± 2.62	0.228 ± 0.012	0.258 ± 0.046	− 28.8 ± 2.09	− 30.90 ± 2.46	90.11 ± 0.98	91.00 ± 0.39
	3 months	220.3 ± 4.16	202.6 ± 4.72	0.284 ± 0.120	0.244 ± 0.0.049	− 26.83 ± 0.746	− 28.66 ± 1.20	88.07 ± 0.76	89.26 ± 1.03

### In vivo studies

To evaluate the neuroprotective potential of the developed QUE-loaded herbosomal formulations, an Alzheimer’s disease-like model was established in rats using oral administration of aluminum chloride at a dose of 100 mg/kg body weight for 35 consecutive days. All animals, except for the negative control group, were subjected to AlCl_3_-induced neurotoxicity to mimic Alzheimer’s pathology. Following induction, animals were divided into seven groups to assess the comparative effects of different formulations. Group 1 served as the negative control and received intranasal (IN) saline only, while Group 2 represented the AlCl_3_-induced positive control. Group 3 received a QUE suspension. Groups 4 and 5 were administered blank herbosomes formulated with Tween 80 and cocamidopropyl betaine, respectively, to assess the potential influence of the carrier systems alone. Groups 6 and 7 received QUE-loaded herbosomes containing Tween 80 (F5) and cocamidopropyl betaine (F6), respectively. This design enabled the evaluation of both the therapeutic efficacy of QUE and the role of the surfactant type in modulating drug delivery and neuroprotective outcomes in an Alzheimer’s disease context. Behavioral testing was performed, followed by biochemical assays to quantify oxidative stress and inflammatory markers. Histopathological analysis was also conducted to assess structural changes in brain tissues. The outcomes were analyzed to determine the relative neuroprotective efficacy of each formulation and to explore the potential of cocamidopropyl betaine as a substitution for Tween 80, aiming to maintain therapeutic performance while improving biocompatibility and safety in herbosomal drug delivery systems.

#### Behavioral test

##### Morris water maze (MWM) and passive avoidance test

**Fig. 6:**
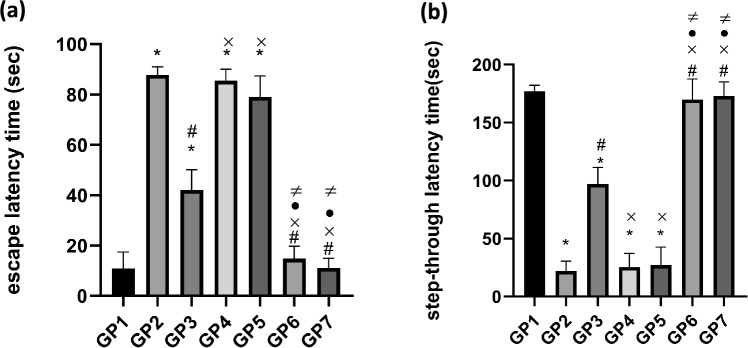
Effect of different QUE formulations on (**a**) Escape latency time (sec) of morris water maze test and (**b**) Step-through latency time (sec) of passive avoidance test in AlCl_3_-induced Alzheimer’s disease rat model. Data are expressed as mean ± SD (n = 6) and were analyzed using one way ANOVA followed by Tukey’s post hoc test. Statistical significance was set at *p* < 0.05. * significant relative to −ve control, # significant relative to + ve control, × significant relative to QUE suspension, •significant relative to blank Tween herbosomes, ≠ significant relative to blank cocamidopropyl betaine herbosomes.

Figure 6a shows that the escape latency time in the MWM test was significantly prolonged in the positive control group compared to the negative control group, demonstrating memory and spatial learning impairment. Similarly, Fig. 6b shows a significant decrease in time spent in the light compartment during the passive avoidance test in the same group, further confirming the cognitive deficits induced by AlCl_3_ administration. Results of QUE herbosomes-treated groups (GP6-GP7) show significant improvement of cognitive and memory performance approaching normal values in both MWM and passive avoidance test compared to control, blank and drug suspension, the consistency between results of both behavioral tests confirm the superior therapeutic potential of the developed QUE-loaded herbosomes in ameliorating Alzheimer disease related memory and cognitive impairment and this may be related to the improved solubility and enhanced BBB penetration ability of the developed Nano formulations. The results of the drug suspension group (Gp3) show significant moderate improvement compared to the positive control group. This improvement may be due to the intrinsic anti-Alzheimer activity of QUE; however, its activity is limited by low aqueous solubility and bioavailability. Blank formulations (Gp4-Gp5) show no improvement in cognitive performance in both behavioral tests, indicating a lack of any therapeutic potential of the vehicle. Overall, the results show the superiority of the developed nano formulations in enhancing therapeutic outcomes and overcoming limitations of conventional dosage, and the findings of both developed formulations support cocamidopropyl betaine as a biocompatible and effective alternative to Tween 80.

#### Biochemical assessment

**Fig. 7:**
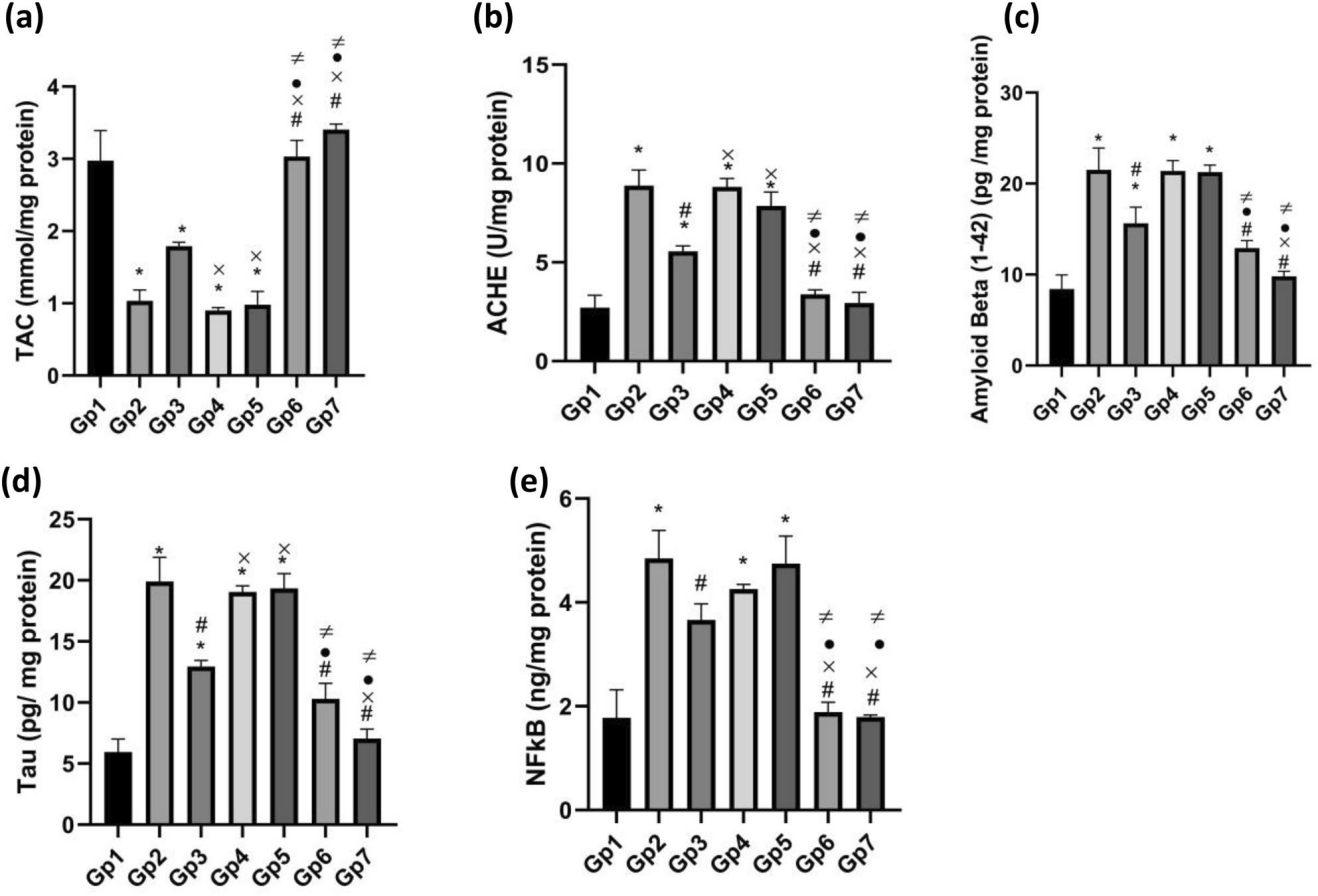
Comparison between the Effect of different QUE formulations on hippocampal (**a**) TAC (mmol/ mg protein), (**b**) AChE activity (U/mg protein), (**c**) amyloid beta (Aβ 1-42), (**d**) tau protein (pg/mg protein) and (**e**) Nuclear Factor Kappa B (ng/mg protein) in AlCl3-induced Alzheimer’s model in rats. Data are expressed as mean ± SD (n = 6) and were analyzed using one way ANOVA followed by Tukey’s post hoc test. statistical significance was set at *p* < 0.05. * signicicant relative to −ve control, # signicicant relative to + ve control, × signicicant relative to QUE suspension, •signicicant relative to blank tween herbosomes , ≠ signicicant relative to blank cocamidopropyl betaine herbosomes

To further evaluate the neuroprotective potential of the developed QUE formulations, a comprehensive biochemical assessment was conducted in the hippocampal tissue of AlCl_3_-induced Alzheimer’s disease rat models. The analysis included key biomarkers associated with oxidative stress, cholinergic function, and neurodegeneration. Specifically, total antioxidant capacity (TAC), acetylcholinesterase (AChE) activity, amyloid beta (Aβ 1–42), Tau protein (PT-181), and nuclear factor kappa B p65 (NF-kB p65)) levels were quantified. These parameters demonstrate the extent of neuronal damage and the therapeutic efficacy of each formulation. Figure 7 illustrates a comparative analysis of these biomarkers across the different treatment groups. In group 2 (Gp2), a reduction in TAC was observed, indicating elevated oxidative stress, which markedly affects brain cells due to their high content of unsaturated fatty acids. This oxidative imbalance contributes to neuronal damage and the emergence of Alzheimer’s disease (AD) markers^58^. Additionally, a significant increase in AChE activity was observed, reducing acetylcholine levels, a crucial neurotransmitter for synaptic communication and cognitive function^59^. The elevated phosphorylated tau protein and Amyloid Beta (1–42) levels are observed, which are considered the most important biomarkers of Alzheimer’s disease pathology, leading to neuronal inflammation and neuronal toxicity, accelerating cell death and declining cognitive functions^60^. An elevated level of Nuclear Factor Kappa B p65, which is involved in the formation of pro-inflammatory cytokines, initiates neuronal inflammation^61^. The results of the QUE herbosome-treated groups (Gp6 & Gp7) demonstrated a marked improvement across all measured parameters, highlighting the neuroprotective potential of QUE via its antioxidant properties, AChE inhibitory activity, and anti-inflammatory effects^62^. Moreover, the optimized QUE herbosomes showed significantly greater efficacy compared to the pure QUE suspension, which may be related to their enhanced ability to cross the BBB owing to the smaller vesicle size^63^. Moreover, Tau protein and Amyloid Beta (1–42) in (Gp7) showed a statistically significant reduction (*p* < 0.05) compared to (Gp3) and (Gp6), suggesting enhanced neuroprotective efficacy of Gp7. This improvement may be attributed to the slightly smaller Vesicle size and lower hydrophilic-lipophilic balance (HLB) value of cocamidopropyl betaine, which favors lipophilicity and potentially enhances permeability across the BBB, thereby improving therapeutic delivery to the brain. QUE suspension group (Gp3) results show significant moderate improvement compared to the positive control group, which may be related to the intrinsic antioxidant, anti-ACHE activity, and anti-inflammatory effect of QUE; however, its activity is limited due to its low solubility and bioavailability. Blank formulations (Gp4-Gp5) show no improvement in biochemical parameters, indicating a lack of any therapeutic potential of the vehicle. Collectively, the results demonstrated the superiority of the developed herbosome formulations in enhancing therapeutic outcomes and overcoming the limitations of conventional dosage. Also, findings of both developed formulations support cocamidopropyl betaine as a biocompatible and effective alternative to Tween 80.

### Histopathological and morphometric examination

**Fig. 8:**
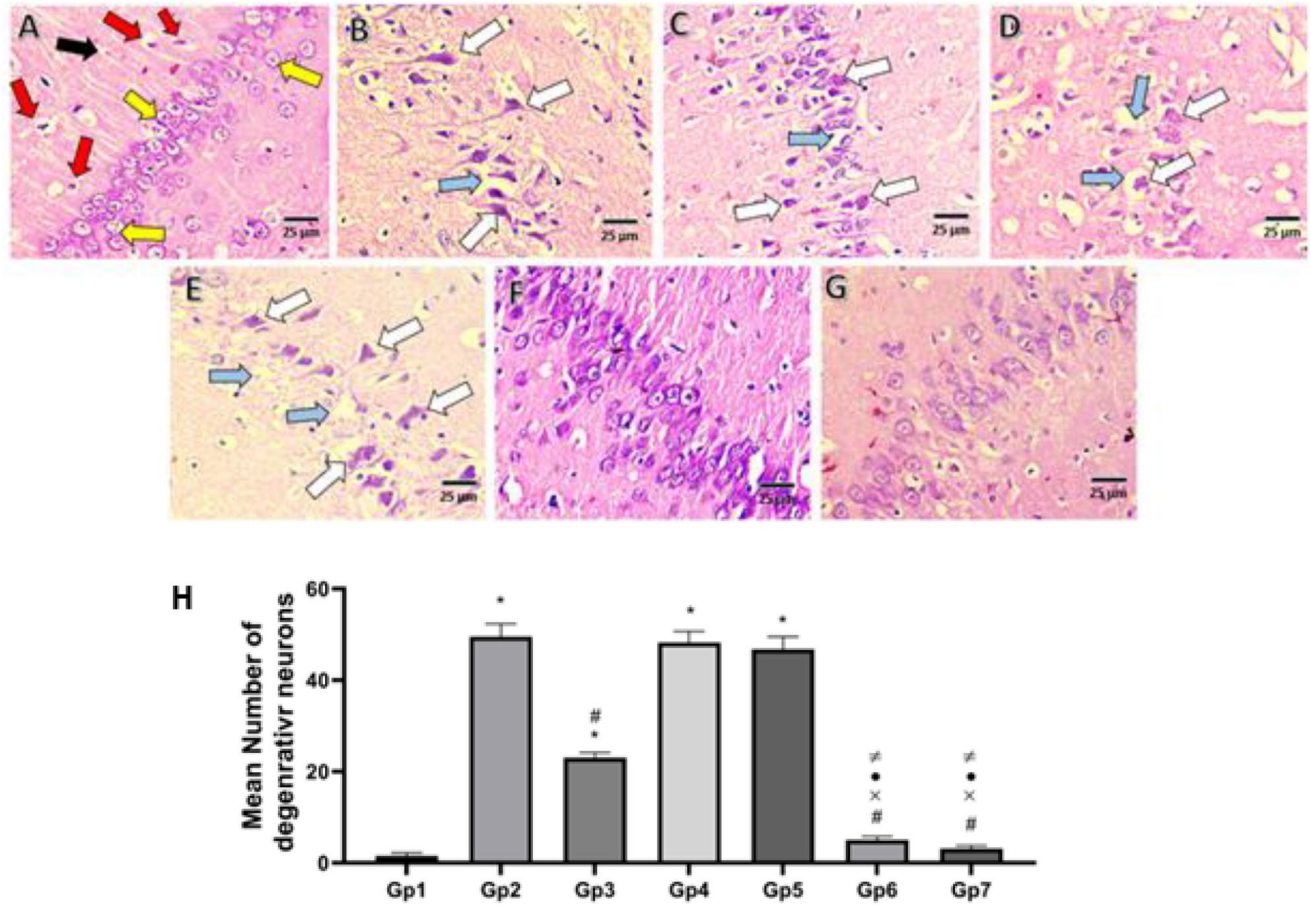
Comparison between the Effect of different QUE formulations on Histopathological changes in Cornu Ammonis zone 1 (CA1) in hippocampus of AlCL_3_ – induced AD model in rats (H&E stained section, 40X with scale bar 25 µm): (**A**) the negative control group; (**B**) AlCl3-induced AD; (**C**) QUE suspension; (**D**) blank Tween herbosomes; (**E**) blank cocamidopropyl betaine herbosomes; (**F**) F5; (**G**) F6 and (**H**) Morphometric analysis of the Mean Number Degenerated Neurons (yellow arrow, normal pyramidal neuron; red arrow, normal glial cells; black arrow, neutrophil; White arrow, shrunken darkly stained pyramidal cells; and blue arrow, vacuolated cells. Data are expressed as mean ± SD (n = 6) and were analyzed using one way ANOVA followed by Tukey’s post hoc test . statistical significance was set at *p* < 0.05. * signicicant relative to -ve control, # signicicant relative to + ve control, × signicicant relative to QUE suspension, •signicicant relative to blank tween herbosomes, ≠ signicicant relative to blank cocamidopropyl betaine herbosomes.

The results of histopathological examination of Cornu Ammonis zone 1 (CA1 zone) of H&E-stained sections Fig. 8A–G) revealed that the − ve Control group (GP1) showed normal pyramidal neurons (yellow arrow) with normal glial cells (red arrow) and neutrophils (black arrow). However, the + ve Control group (GP2) showed pathogenic abnormalities, having a large number of shrunken, darkly stained pyramidal neurons (white arrow) and vacuolated cells (blue arrow). Treatment with Blank formulations (Gp4, Gp5) showed no improvement in histopathological abnormalities associated with the positive untreated group. Treatment with free QUE suspension in (Gp3) showed a moderate improvement in histopathological abnormalities compared to the positive control group. In contrast, the results of the QUE herbosome-treated groups (Gp5, Gp6) demonstrated a significant attenuation of these histopathological abnormalities. In addition, Morphometric examination of the mean number of degenerative neurons shows a significantly higher number of degenerative neurons in the AlCl3-induced AD-model group compared to the negative control group (Fig. 8H). Interestingly, administration of QUE herbosomal formulations (F5-F6) resulted in a significant reduction in the mean number of degenerative neurons compared to the positive control group or free QUE suspension. Collectively, these results showed the superiority of the developed formulations in restoring the hippocampal Cornu Ammonis zone 1 histological pattern with significantly higher efficacy than free QUE suspension. Also, findings of both developed formulations support cocamidopropyl betaine as a biocompatible, effective alternative to Tween 80.

### Study limitations

The findings of this study should be interpreted considering certain limitations. First, the AlCl3-induced AD model used in the current study reflects acute, partially reversible neurotoxicity rather than progressive chronic pathology shown by a 5xFAD or a 3xTG transgenic mouse models. Consequently, while this model is suitable for evaluating neuroprotective effects and formulation performance, it may not fully mirror the chronic progression of AD.

Second, the study was primarily designed to evaluate formulation efficiency, brain targeting potential, and neuroprotective mechanisms of QUE-loaded herbosomal nanocarriers; therefore, comprehensive longitudinal metabolic monitoring and SHIRPA-equivalent behavioral phenotyping were beyond the scope of the current study. The behavioral assessments conducted were exploratory and formulation-focused by design, serving as an indicator of functional benefit. Accordingly, comprehensive behavioral phenotyping constitutes a well-defined next phase in the development and translational validation of this herbosomal nanocarrier platform and will be addressed in future dedicated studies.

## Conclusion

QUE herbosomes stabilized with either the synthetic non-ionic surfactant Tween 80 or the natural-origin surfactant cocamidopropyl betaine were successfully prepared via the thin-film hydration technique, yielding nanocarriers with favorable physicochemical characteristics. Both optimized formulations demonstrated pronounced neuroprotective activity in an AlCl_3_-induced Alzheimer’s disease rat model, as evidenced by improved learning and memory performance, reduced hippocampal tau and Aβ protein accumulation, and decreased acetylcholinesterase activity. A comparative evaluation showed no statistically significant differences in therapeutic activity between the optimized formulations (F5, F6) across the measured parameters. These findings underscore cocamidopropyl betaine as a biocompatible, safe, and efficacious alternative to synthetic surfactants, with potential to advance the development of sustainable nanocarrier platforms. Such systems may improve therapeutic efficacy in Alzheimer’s disease and could be extended to the management of other neurodegenerative disorders driven by oxidative stress.

## Data Availability

Data will be made available on request.
